# Help Seeking Behavior of Women with Self-Discovered Breast Cancer Symptoms: A Meta-Ethnographic Synthesis of Patient Delay

**DOI:** 10.1371/journal.pone.0110262

**Published:** 2014-12-03

**Authors:** Zohreh Khakbazan, Ali Taghipour, Robab Latifnejad Roudsari, Eesa Mohammadi

**Affiliations:** 1 School of Nursing and Midwifery, Mashhad University of medical science, Mashhad, Iran; 2 Health Sciences Research Center, Department of Epidemiology and Biostatistics, School of Health, Mashhad University of Medical Sciences, Mashhad, Iran; 3 Evidence-Based Care Research Center, Department of Midwifery, School of Nursing and Midwifery, Mashhad University of Medical Sciences, Mashhad, Iran; 4 Medical Sciences Faculty, Nursing Department, Tarbiat Modares University, Tehran, Iran; University of Stirling, United Kingdom

## Abstract

**Background and Objective:**

Patient delay makes a critical contribution to late diagnosis and poor survival in cases of breast cancer. Identifying the factors that influence patient delay could provide information for adopting strategies that shorten this delay. The aim of this meta-ethnography was to synthesize existing qualitative evidence in order to gain a new understanding of help seeking behavior in women with self-discovered breast cancer symptoms and to determine the factors that influence patient delay**.**

**Methods:**

The design was a meta-ethnography approach. A systematic search of the articles was performed in different databases including Elsevier, PubMed, ProQuest and SCOPUS. Qualitative studies with a focus on help seeking behaviors in women with self-discovered breast cancer symptoms and patient delay, published in the English language between 1990 and 2013 were included. The quality appraisal of the articles was carried out using the Critical Appraisal Skills Programme qualitative research checklist and 13 articles met the inclusion criteria. The synthesis was conducted according to Noblit and Hare’s meta-ethnographic approach (1988), through reciprocal translational analysis and lines-of-argument.

**Findings:**

The synthesis led to identification of eight repeated key concepts including: symptom detection, initial symptom interpretation, symptom monitoring, social interaction, emotional reaction, priority of medical help, appraisal of health services and personal-environmental factors. Symptom interpretation is identified as the important step of the help seeking process and which changed across the process through active monitoring of their symptoms, social interactions and emotional reactions. The perceived seriousness of the situation, priority to receive medical attention, perceived inaccessibility and unacceptability of the health care system influenced women’s decision-making about utilizing health services.

**Conclusion:**

Help seeking processes are influenced by multiple factors. Educational programs aimed at correcting misunderstandings, erroneous social beliefs and improving self-awareness could provide key strategies to improve health policy which would reduce patient delay.

## Introduction

Breast cancer is the most common cancer and cause of death from cancer in women worldwide [1]. The World Health Organization introduced early detection of breast cancer as “the cornerstone of breast cancer control” [2]. Mammography screening is the most effective method for the early detection of breast cancer [2], [3], but women’s use of mammography is low [4], [5], especially in low income countries [5]. The majority of breast cancer cases are identified in symptomatic women [4], mainly by the women themselves [6], and outside of a structured breast self-exam [7]. However, 20–30% of women delay seeking help for three months or more after the discovery of a breast cancer symptom [8].

There is a strong relationship between patient delay and survival [9], [10], and this has encouraged researchers to identify predictive factors of delay that could inform development strategies in order to reduce patient delay [10]–[12]. In this regard, numerous studies with different methodological approaches have been conducted and these have led to different consistent or inconsistent results; although they have not provided enough information to develop effective policies on this subject [10]–[12].

Systematic reviews are known to be a key source of knowledge in the development of evidence-based practice and policy making [13]–[15]. These studies focus on combining [15], aggregating [15], [16] and summarizing [16], [17] the primary findings of quantitative studies. Systematic reviewers with a commitment to explicit and rigorous guidelines when conducting their studies, facilitate the acquisition of rigorous data for health intervention and decision making [16], [18]. These studies cannot satisfy all of the scientific evidence needed by policymakers [16], [18], however, it seems that increased number of qualitative research in health care domains has attracted the attention of policy-makers to use qualitative research findings as a source of knowledge for evidence-based health policy making [15], [19].

Methods for the secondary synthesis of qualitative research, unlike quantitative methods, have not yet been well developed, and they are also associated with philosophical [20] and methodological challenges [17],[19],[20]. Philosophical assumptions concerning qualitative studies indicate that qualitative findings are contextually based and related to social and cultural issues, thus integrating primary data may be contrary to this unique feature of qualitative studies [15]. In addition, the presence of differing qualitative research methods caused some practical problems for integration, synthesis and generalization of the qualitative findings [15], [17], [20].

However, some researchers have recognized the value of secondary synthesis and believe that generalization across studies adds to the findings of individual studies [15], [19], and they have suggested various methods for the synthesis of qualitative research [21], [22]. Unlike conventional systematic reviews, the synthesis of qualitative studies beyond aggregating and summarizing data, leads to the interpretation and development of existing concepts and theories [16]. In addition, it can guide researchers when conducting future research by illuminating a knowledge gap or saturation [19].

Meta-ethnography is a method for the synthesis of qualitative studies with an interpretive approach introduced by Noblit and Hare (1988) in the field of education [22]. It was then used in the field of health care, especially in topics related to women’s experiences [19]. This method refers to the identification of major concepts and metaphors, determining how these are related, translating them to each other, and building a new understanding, interpretation or theory, rather than just putting together elements of the original papers [20], [21].

Noblit and Hare (1988) have described seven phases in conducting a meta-ethnography [22]. This method is often appropriate for the synthesis of small numbers of qualitative studies [14], [16] and subjects with close conceptual similarity to each other [15], [16].

There has been increasing numbers of qualitative research conducted in the help seeking behaviors of women with self-discovered breast symptoms, and so we aimed to synthesize the existing relevant studies in order to model the factors that influence patient delay, by adopting an interpretive meta-ethnographic approach.

## Methods

**Figure 1:**
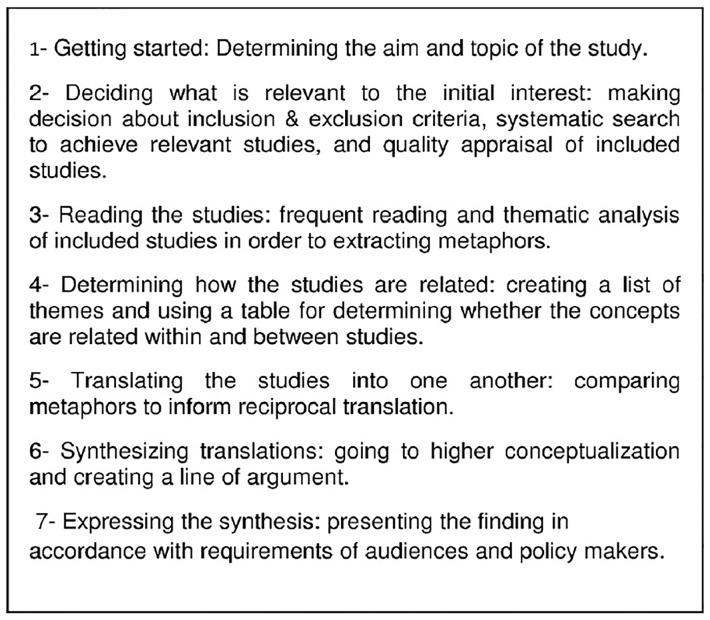
Stages of study according to Noblit and Hare (1988).

This study was performed according to the meta-ethnographic approach, which is detailed by Noblit and Hare (1988), in seven phases (Figure 1).

### Inclusion criteria

In this review, qualitative studies that focused on help seeking behaviors in women with self-discovered breast cancer symptoms and patient delay, written in the English language, and published between 1990 and 2013 were included. Studies with a quantitative approach, lack of adequate focus on patient delay, or identification of symptoms by a doctor or screening procedures were excluded.

### Search strategy

**Figure 2:**
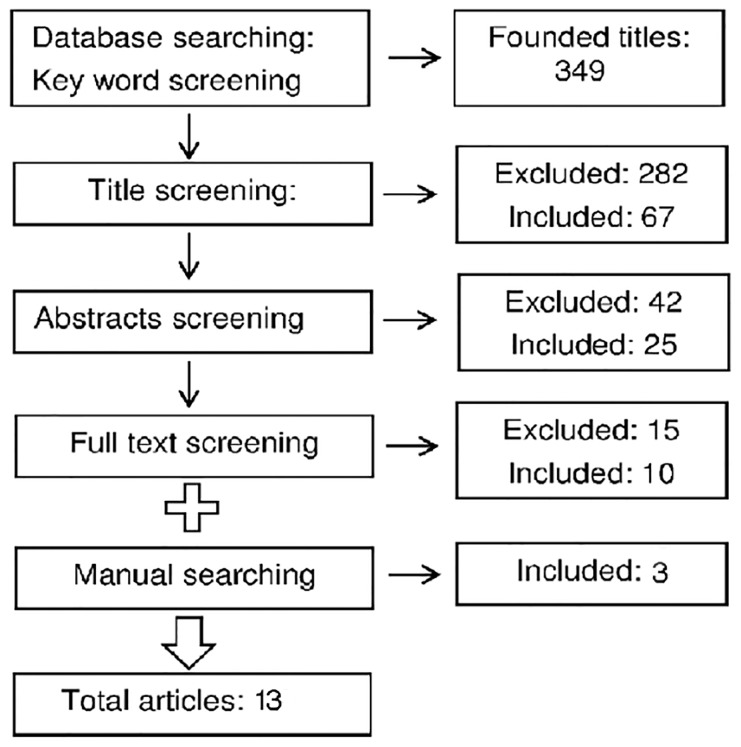
The screening and selection process of the articles.

Search of the articles was performed in a systematic manner using different databases including Elsevier, PubMed, ProQuest and SCOPUS, using the key words: breast cancer, qualitative research, patient delay, help seeking behavior, and health seeking behavior. In addition, a manual search was performed on the references of entered articles. Studies selected were based on a multi-stage screening of key words, titles, abstracts, and the full text of the articles, respectively, to ensure the relevance of the entered articles. In total, 352 papers were identified by a database [349 papers] and manual searching [3 papers] of the journals. A number of studies were excluded at each stage if they were either not relevant to the subject or if they had not used a qualitative approach. Finally, 25 papers entered full text screening. This stage led to selecting 10 qualitative relevant papers from electronic searching. These 10 papers plus three eligible papers obtained through manual searching were appraised using CASP tool. Key word, title, abstract and full text screening were performed by RLR and ZKh. The screening process is illustrated in Figure 2 and the summary of the final articles included in the study is shown in Table 1.

**Table 1:** The summary of final articles included in the study.

Author(s)/Country	Aim	Sample size/Participants’ characteristics	Methods	Setting
Adam and Soutar/Australia (2003) [6]	To provide insight into the process underlying the formation of intentions to seek a medical evaluation for self-discovered breast changes	13 women aged 18 years or over who had received a medical diagnosis for self-discovered breast changes within the previous five years	Qualitative study/Individual in-depth interviews/purposive sampling method	Women’s homes
Unger-Saldana, & Infante-Castaneda/Mexico City (2011) [11]	To develop an understanding of the dimensions involved in delayed medical care of BC patients from their own perspective	17 women with breast symptoms (highly suspicious breast cancer diagnosis) & 11 of their relatives, before the patients’ first consultations with the breast specialist	Qualitative study/Theory of illness behaviour/Purposely selected/in depth interview/Theoretical saturation/Grounded theory approach	The Mexican National Cancer Institute
Lam et al./Hong Kong (2009) [12]	To describe help-seeking patterns in Chinese women with self-detected breast symptoms	37 women with self-detected breast symptoms and age>21 years/Women with a prior BC diagnosis, or whose breast abnormality was discovered through breast screening were excluded	A grounded theory-based qualitative study/Semi-structured interview at first consultation before diagnosis/Theoretical sampling/Grounded theory approach	Three regional Hong Kong public hospitals
Burgess et al./London (2001) [23]	To explore the factors that influence GP consultation by women with BC symptoms	46 women with newly diagnosed breast cancer/Non-delayers = 15 n/delayers (>12 week) = 31 n	Qualitative study/Purposive selection/Semi-structured, open-ended interview/Framework method analysis	Medical oncology outpatients
de Nooijer/Netherlands (2001) [24]	To investigated factors influencing the process of detecting cancer symptoms and seeking medical help	Total of patients = 23 nBreast cancer = 7 nTesticular cancer = 5 nColon cancer = 6 nMelanoma = 6 n	Qualitative study/Andersen’s model/Semi-structured, in-depth interview/Framework analysis	Patient’s homes
O’Mahony/Ireland (2011) [25]	to explore women’s health seeking behaviours for a self-discovered breast symptom	10 women with self-discovered breast symptoms	Qualitative descriptive approach/“Help seeking behaviour & Influencing Factors” framework/Semi-structured interview/content analysis	Women’s homes, the Researcher’s workplace, Telephone interview
Gates et al./Mid-South metropolitan area (2001) [26]	To describe caring behaviours and demands and to identify possible relationships between caring demands and delay for African American women	13 African American women with a primary diagnosis of breast cancer (ages 30–66)	Focused ethnographic design/Audiotaped ethnographic interviews, snapshots, participant observation/Purposefully selected/Leininger’s phases of ethnographic analysis	Two oncology clinics in the mid-South
Lackey et al./Mid-South metropolitan area (2001) [27]	To describe the experiences of African American women living with breast cancer following the primary diagnosis	13 African American women/between 30 and 70 years of age, within three to six months of diagnosis, and initial treatment	Phenomenology/Purposefully selected/Colaizzis analysis plan	Two oncology clinics in the large mid-Southern metropolitan area
Lu et al./Taiwan (2010) [28]	To explore the experiences of older Taiwanese women when they first faced a new diagnosis of breast cancer	14 women, aged 65 to 91 years with a new diagnosis of breast cancer	Qualitative research design/Purposive sampling/In-depth interview/Content analysis	Cancer hospital in northern Taiwan
Dye et al./Ethiopia (2012) [29]	To assess the initial experiences, symptoms, and actions of patients in Ethiopia ultimately determined to have breast cancer	Total Sample = 69 n including 55 patients & 14 proxies/Mean time ignored = 1.6 years	Mixed qualitative and quantitative approaches/Semi structured interview/Theme analysis	The main national cancer hospital
Taib, et al./Malaysia (2011) [30]	To explore the experience of Malaysian women presenting with advanced breast cancer with regards to their interpretation of breast symptoms	19 breast cancer patients presenting with delayed treatment and/or advanced cancer	Grounded theory/Purposive sampling/In-depth interviews/Thematic analysis	Outpatient clinics & in-patient admissions at the University Malaya Medical Centre
Rastad et al./Iran (2012) [31]	To gain insight into the causes of delay in seeking treatment in patients with breast cancer	10 breast cancer patients in the stages of II b, III or IIV, with at least 3 months patient delay in seeking treatment	Qualitative study/Semi-structured interviews/content analysis	A major oncology clinic in Kerman, Iran
Mathews et al./North Carolina (1994) (32)	To explore the factors that contribute with presentation for treatment with advanced stage disease	26 black women who presented with late-stage, advanced breast tumors(defined as TNM stage 3 or greater)	Qualitative study/In-depth interviews/Narrative analysis	ECU (East Carolina University) breast clinic and patient’s home

### Quality appraisal

Although quality appraisal is an important step in a conventional systematic review [16], [19], there is little consensus on the need and essential criteria for conducting it in a qualitative meta-synthesis [15], [16], [19], [32]. Some researchers believe that a qualitative appraisal is necessary in order to eliminate poor studies. But others state that weak studies make a lower contribution in the analysis and are eliminated naturally [32]. Some challenges regarding the use of quality appraisal have been raised because of the possibility of losing relevant studies [15], [19] and giving priority to relevancy, rather than the methodology of the study [16], [33]. However, although using quality appraisal tools may not be a necessary element of such studies, it does provide an identical format for reviewers [34] and this could facilitate the identification of any weaknesses when interpreting the findings [19]. Noblit and Hare (1988) described “quality in terms of quality of metaphors”… [21]. However more recent meta-ethnographic researchers have used a modified version of CASP [19], [35], [36], [37].

In this review we decided to assess all included papers)13 full text articles(in order to determine the contributions of each paper to the synthesis (relevance) and to describe the methodological quality of the included papers according to their strengths and weaknesses using modified version of CASP tool [38]. Appraisal was independently carried out by two reviewers and consensus was achieved through discussion. Assessing relevance revealed two groups of papers according to their contribution to the synthesis: perfectly relevant [6], [11], [12], [23]–[25], [27], [31] and relatively relevant articles [26], [28]–[30], [32]. Perfectly relevant articles were completely focused on help seeking and delay in breast cancer, whereas in relatively relevant studies help seeking or delay in breast cancer was only one of the focused concepts rather than the core focus of the article. Both groups were considered eligible to be included in the methodological appraisal. Then based on ten questions of the CASP tool, the quality of the 13 included studies was assessed (Table 2). All of the studies possessed a favorable quality score, and no study was rejected based on this appraisal tool. In all studies, the research objectives, sampling strategy and data collection methods were clearly described. In the majority of studies, reflexivity (reflection of researchers on their own thoughts and ideas and their relationships with participants) was not clearly described. In all studies, ethical considerations were described without details. In the majority of studies, the analytical methods were reported, but they lacked an in-depth description of the analysis process. All studies described their research findings explicitly, although in some cases they had an inadequate in-depth description. Nearly all studies discussed their findings in relation to the relevant literature.

**Table 2:** Quality appraisal of included studies (modified from CASP criteria (CASP, 2006).

Are the followingclearly described?	Lam et al.[12]	Burgess et al.[23]	Unger- Saldana, InfanteCastan[11]	de Nooijer [24]	O’Mahony [25]	Gates et al. [26]	Lackey et al. [27]	Lu et al. [28]	Adam and Soutar [6]	Dye et al. [29]	Taib, et al. [30]	Rastad et al. [31]	Mathews et al. [32]
Research questions	+	+	+	+	+	+	+	+	+	+	+	+	+
Appropriateness of qualitativeresearch methodology	+	+	+	+	+	+	+	+	+	+	+	+	+
Research method	+	+	+	+	+	+	+	+	+	+	+	+	–
The recruitment strategy	+	+	+	+	+	+	+	+	+	+	+	+	+
Data collection	+	+	+	+	+	+	+	+	+	+	+	+	+
Reflexivity	+	–	–	–	–	+	+	+	–	–	+	+	–
Ethical consideration	+	+	+	+	+	+	+	+	+	+	+	+	+
Rigorous analysis	+	+	+	+	+	+	+	–	+	–	+	–	+
Findings	+	+	+	+	+	+	+	+	+	+	+	+	+
Contribution to knowledge	+	+	+	+	+	+	+	+	+	+	+	+	+

To ensure the validity and reliability of the data extraction and analysis, it was endeavored to have constant comparison of data extracted and also shared discussions with other authors in different analytical steps.

### Synthesis

Meta-ethnography, based on the Noblit and Hare approach [1988], focuses on three analysis methods including reciprocal translational analysis (RTA), reputational synthesis and lines-of-argument [21], [22]. Reciprocal translational analysis refers to the determination of metaphors and concepts in each study, comparing them with other studies and choosing one concept that encompasses other similar concepts. Reputational synthesis involves identifying the divergence between studies and rationalizing it [22]. Lines-of-argument refers to building a picture of relationships between the main concepts and going beyond the description [15], [22], to develop a “whole” [22], theory, model, or new understanding of the subject under study [34].

In this review, in order to translate the concepts to each other (RTA), the full text of the included articles was read repeatedly, and themes of individual articles were extracted through thematic analysis. Then a large table was drawn, in which each column was allocated to one study’s extracted concepts, and each row was devoted to the similar concepts extracted from all articles. One article was considered as an index study [11], which provided wider conceptual coverage for comparing the extracted concepts. Codes that evolved from this article and other articles were constantly compared with each other. This required frequent references to the original articles. Constant comparison across and between studies led to evolving identical concepts, conceptually similar meanings, new concepts, and infrequently concepts with opposite meanings. To translate the conceptually similar themes, one overarching concept that provided better coverage for the other concepts, was adopted and modified or developed by a constant comparison method during the review. We outlined themes and sub-themes in one table [Table 3].

**Table 3:** Extracted themes and sub-themes through reciprocal translational analysis.

Themes	Subthemes/Triggers to seeking help	Subthemes/Barriers to seek help
**Symptom detection**	Detection of a lump [12], [23], [5], [27], detection of painful lump[6], [11], [12], [23], [24], [26]–[30], [32] and visible symptoms like skinchanges and breast discharge [11]	Detection of painless lump [11], [12], [23], [24], [28]–[32], detection of a non-lump breast symptom (23)
**Initially symptom** **interpretation**	Attribution of symptoms to serious conditions/cancer = Cancer knowledge and matching symptomswith women’s knowledge regarding breast cancer symptoms[11], [12], [23]–[25], women’s perception of being at risk of BC[6], [11], [24], [25], [28], [30]	Attribution of symptoms to non-serious conditions = Non-matching symptoms with women’s knowledge regarding BC symptoms [11], [12], [23]–[25], [30], [31], women’s perception of not being at risk of BC [6], [11], [24], [25], [28], [30], [31], past history of benign diseases of breast [12], [23], [26], [27], [30], [31], lack of family history [27], [30], some general beliefs [11], [31], discovering breast changes during pregnancy [30]
**Symptom monitoring**	Persistent symptoms [11], [12], [32], symptom development[12], [23], [24], [29], [30], enlarged size of the breast[12], [24], [28], the appearance of pain [12], [23], [24], [28]–[30], [31], [32]and physical discomfort interference with daily activities [11], [32]	Fluctuation in symptoms [12], [30], [32]
**Emotional reactions to** **symptoms**	Concerns about the nature of the symptoms [11], [24], [25],fear about the consequences of delay [12], [24]	Lack of worry about the nature of the symptoms [6], [12], [24], [26], [29], [30], fear of cancer diagnosis [11], [12], [24], [30], [31], fear of cancer as an incurable disease [11], [12], [24], [25], [28], [30], [31], [32], fear of the consequences of medical help-seeking [11], [12], [23], [26], [28], [32], denial [11], [24], [25], [30], [31], fear of losing femininity [27]
**Social interactions**	Receiving various kinds of social support following symptom’s disclosure:financial support [11], [27], informational support to interpret symptom [12], [24],advice to seek treatment [11], [12], [23], [24], reducing concerns [11], [28],being under pressure of others to seek help [12], [29], [30]	Receiving wrong information, and suggestions based on waiting policy [11], [26], [30], lack of partner support [26], concern about being a burden on family [30]
**Priority of medical help**	Perceived need to confirm the diagnosis [6], [12], [23], [24], prior regularattendance to GPs for health problems [23]	Alternative therapy before medical help seeking [28], [30], competing priorities [6], [11], [12], [23], [25], [30], [31], lack of trust in conventional treatment [30]
**Appraisal of** **health services**	Bing confident to share the problem with health professionals [23]–[25]	Lack of insurance services [11] and financial constraints [6], [11], [12], [25], difficult access to health care services [11], [24], [25], [30], unawareness of breast clinics location [12], lack of women’s trust to knowledge and skills of physicians [24], [25], unpleasant experiences related to the health services providers [11], shame and embarrassment of breast examination [12], [28], concerns about unnecessary presentation or bothering physician [6], [23], [24], women’s preferences to be visited with a female GP [25], frustrating referral process [11]
**Personal-environmental** **factors:**	Positive view about curability of cancer [25], spiritualty [27]	Low socioeconomic status [11], recently immigrated [12], older women [12], [28], general beliefs about disease e.g., “your body has no pain it means that there is no injury” [11], “a wound which opens up, and heals by itself, little by little [31], “if a lump ain’t bothering you, you shouldn’t bother it. It will probably go away” [32], optimistic beliefs related to the nature of symptoms (30), fatalistic beliefs (25, 30, 32)

**Figure 3:**
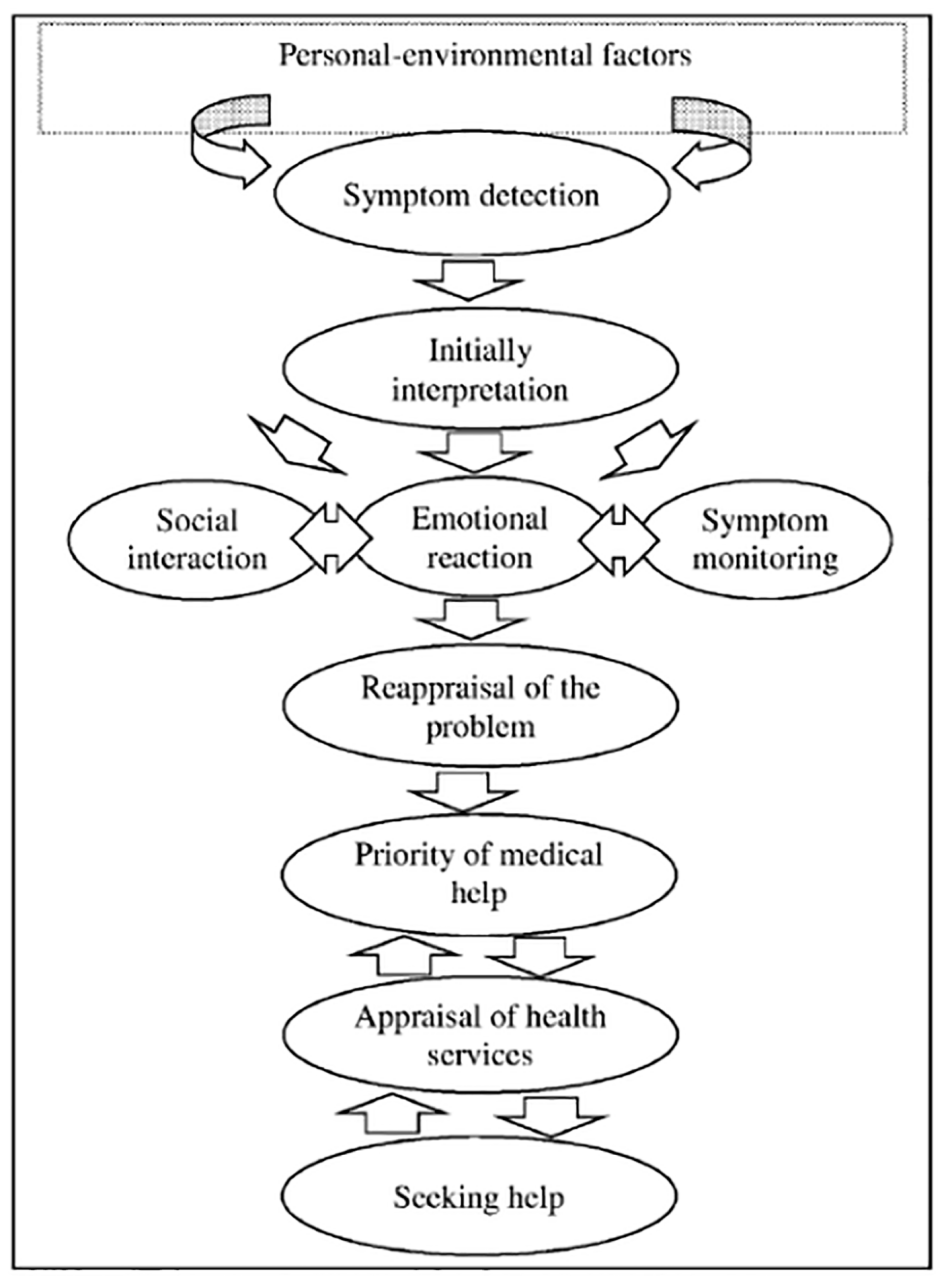
Model of help seeking process and influencing factors.

Ultimately, lines-of-argument was conducted through establishing linkages between the main concepts that emerged from the reviewed studies to develop a model that could predict help seeking processes in women with self-discovered breast symptoms (Figure 3).

## Findings

The synthesis in the reciprocal translation stage resulted in emergence of eight key concepts that were repeated in the literature. These concepts included: symptom detection, initial symptom interpretation, symptom monitoring, social interactions, emotional reactions, priority of seeking medical help, appraisal of health services and personal-environmental factors.

### Symptom detection

In the reviewed studies, women detected different symptoms including lumps [6], [11], [12], [23], [24], [26]–[30], [32] which were the most frequently detected symptom, pain [11], [12], [23]–[25], [30], and discharge from the nipple [11], [24], [25], inverted nipple [24], [31], axillary mass [31], arm pain [32] and weakness and dizzy [32]. The patient’s delay from the detection of symptoms to seeking help varied from a few days [11], [23], [25], [27] to several years [11], [29], [30]. Women mostly detected these symptoms by chance, [31], [30], [28], [29], [24], [12] for instance while having a shower [25], [27], [31], when getting dressed or during breastfeeding [25]; although in some cases, the symptoms were discovered by active detection through a breast self-examination [6], [27], [25], [23], [24].

### Initially symptom interpretation

Symptom interpretation was identified as the first [6], [11], [12], [23] and the most important step of the help seeking process after symptom detection [12], [23], [24], [30]. At this stage, women assessed and attributed their symptoms to a cause and labeled it as a normal, ambiguous, or serious condition [11], [12], [23], [24], [30], [32]. Women initially tended to interpret the breast symptom as a normal or non-life-threatening condition [11], [12], [24], [26], [28], [29] such as hormonal changes [6], [12], trauma [12], [28], [32] or breastfeeding [28], [30]. nterpreting the symptoms as normal changes, less serious [6], [11], [12], [23], [24], [26], [29]–[32] or ambigus [6], [12], [25], [30] conditions contributed to patient delay.

Initially symptom interpretation was mainly influenced by interacting of issues such as nature of the symptoms [11], [12], [23], [24], [29], women’s knowledge [11], [12], [23]–[25], [30], [31] and women’s perception regarding being at risk of breast cancer [6], [11], [24], [25], [28], [30], [31].

A painless lump tended to be perceived as a non-serious condition in studies with different nationalities including Mexico [11], China [12], Malaysia [30], Ethiopia [29], Iran [31], Taiwan [28] and black women in North Carolina [32]. However, in one study, the experience of a non-lump breast symptom was a common cause of delayed presentation.

Symptoms that were compatible with the women’s knowledge and expectation of breast cancer symptoms usually resulted in action [12], [23], [24]. In contrast, symptoms that were perceived as a common ailment [24] or were not compatible with the women’s expectation of breast cancer symptoms were interpreted as less serious or ambiguous changes [12], [23].

Previous experience of benign breast disease [12], [23], [26], [27], [30], history of cancer in the family [12], [24], [26], [27], and knowing people who suffered from cancer in relatives or friends, not only influenced knowledge and interpretation of symptoms, but it also affected the women’s decision making process [11], [12], [24]–[27].

Some studies in our synthesis also showed that the women’s perception of being at risk of breast cancer had a great impact on symptom interpretations, so that often women who considered themselves at low risk for breast cancer attributed their symptoms to less serious causes and vice versa [6], [11], [24], [25], [28], [30].

A family history of cancer had different and conflicting effects on the perceived risk of breast cancer [11], [12], [25], [26], [31] and shaped positive/negative beliefs and experiences about the curability of the disease [11], [12], [30].

Generally, women with a family history of breast cancer had a greater awareness of breast cancer [25], considered themselves at higher risk and experienced greater fear in dealing with symptoms [11], [12], [25], [26], [31], and women who did not have a family history of breast cancer, perceived themselves at low risk and delayed seeking help [25], [30]. However prior negative experiences about the curability of breast cancer deterred some women from seeking help [11], although in contrast it prompted others to seek help as soon as possible [11], [25].

### Symptom monitoring

The initial interpretation of symptoms changed across the help seeking process through monitoring of symptom progression and information seeking via social interactions. Symptom disclosure and receiving social signals often were used by patients to evaluate, confirm or develop their own interpretations [11], [12], [23], [24], [30].

Women, who initially dismissed the breast symptom, often monitored their symptoms for change and kept them under monitoring [12], [23], [30]. In most cases, persistent symptoms [11], [12] and symptom development [12], [23], [24], [29], [30], [32] such as enlarged size of the breast [12], [24], [28] and visible symptoms like skin changes and breast discharge triggered women to reappraise their initial interpretation and motivated them to seek help. The appearance of pain [12], [23], [24], [28]–[30], [31], [32] and physical discomfort, especially if they interfered with women’s daily activities [11], [32], were the most common triggers to action in several studies with different cultural contexts.

### Social interactions

In most of the reviewed articles, social interaction was an influencing factor on the help seeking process. Disclosing the discovered symptoms was a repeated concept in most reviewed articles, and had more positive than negative effects on help seeking behaviors [11], [12], [23]–[26], [28]. Although some women did not disclose their symptoms to anyone else, due to concerns such as bothering others [11], [24], [25], most of the women talked about their symptoms with a lay person and received various kinds of social support [11], [12], [23]–[25].

The majority of the reviewed studies focused only on the supportive role of symptom disclosure [12], [23], [24], [25], [28], [29], and other studies also noted the negative effect of discussing with others in some cases [11], [26], [30], [31].

Symptom disclosure to others had supportive role to interpret symptom [12], [24], [30]. Some women, who had initially ignored the breast symptom, reinterpreted the symptom differently following getting new information; for instance an alert message implied required attention and provoked help seeking [11], [12], [24], [30], while receiving misinformation and reassurance messages that implied a “wait-and-see approach” could act as a barrier to seeking help [11], [26], [30], [31]. These later messages were more evident in the participants’ narrative of Malaysia [30] and Iran [31], as lay persons had low scientific knowledge and experience about breast cancer.

Indeed, women received other kinds of social support. For example family and other relatives attempted to provide women with their emotional support [11], [28], and financial support [11], [27], as well as to reassure [11], encourage, and advise women to seek treatment [11], [23], [24]. In some cases, the pressure bought to bear from others (spouse, relatives and colleagues) resulted in medical seeking [12], [29], [30].

Tiab et al (2011), stated that, “The need for being sanctioned sick or ill was an important trigger to label the symptoms as serious. Therefore, when significant other individuals had no information about signs of cancer, the symptoms were left unattended” [30] **.**

Receiving messages via social media could also influence women’s interpretation of their symptoms [12], [24], [30] and also their decision making process of help seeking [23], [24], [30]. Multimedia resources were an important source of information in Chinese women [12].

### Emotional reactions to symptoms

Emotional reactions after discovering symptoms were nearly highlighted in all of the reviewed studies. While women who did not perceive the seriousness of the symptom, might dismiss their symptoms without any emotional reaction [6], [12], [24], [26], [29], [30], nearly all of the women who recognized the seriousness of the symptoms experienced different types of emotional feelings, such as; anxiety, uncertainty [19], [24], [26], depression [25], [28], hopelessness [28], and various forms of fear.

These psychological responses often caused conflicting outcomes in the help seeking process [11]. According to one study, “fear seemed to provoke one of two opposite actions in the women that experienced it: delayed seeking of medical attention to avoid confirmation of a cancer diagnosis, or acceleration of medical help-seeking to receive treatment as early as possible” [11]. Emotional reactions and the way women respond to them (prompt or delayed help seeking), appear to be related to the nature of the symptoms [23], [24], the women’s prior experience of cancer in relatives [11], [23], and their perceived risk of cancer [11].

Although the impact of various types of fear on the help seeking process was unclear, perceived seriousness of the symptom and fear about the consequences of delay such as fear of death; motivated women to seek help [24]. Overall, a lack of concern about the nature of the symptoms [24], denial [11], [24], [25], [30], [31], fear of confronting a cancer diagnosis [11], [12], [24], [30], [31], fear of cancer as an incurable disease [12], [24], [30], [32], and fear of the consequences of treatment, including breast surgery [11], [12], [23], [26], [28], [32], caused delayed help seeking. Fear of loss of femininity was only reported by Lackey et al. [2001], in African American women following a diagnosis of breast cancer [27].

Denial as a common coping strategy was influenced by symptom progression; therefore, among the women who delayed seeking help due to denial in accepting the possibility of cancer, the development of pain or nipple discharge provoked action [11].

### Priority to medical help

Most of the women, who understood the seriousness of their condition, tried to seek medical care to confirm the diagnosis [6], [12], [23], [24] and some women that attributed their symptoms to less serious causes or those who were frightened of medical confirmation, initially sought alternative therapy as a simple way to deal with the symptoms before medical help seeking [28], [31]. Some of the Malaysian women, due to uncertainty about the ability of conventional medicine to combat the fatal outcomes of cancer, chose alternative medicine [30]. Some women applied alternative therapies as an adjunctive treatment for decreasing the adverse effects of conventional treatment [24], [28]. However, alternative therapy was taken less into account by the authors of the reviewed articles.

Some women, despite their understanding of the potential seriousness of their symptom, had delayed symptom presentation because of occupational or family commitments [6], [11], [12], [23], [25], [31]. Competing priorities were significant barriers for help seeking in these women which led to giving higher priority to others rather than their own health needs. Whereas, in African American women, caring behaviors towards family and others did not play a negative role [26].

### Appraisal of health services

Finally, women made a decision about the source of care and appraised the feasibility of using health services in an implicit manner**.** Financial constraints [6], [11], [12], lack of insurance services [11], difficulty accessing health care services for a number of reasons such as distance [11], [30] and lack of knowledge of breast clinic locations [12] were identified as factors that impeded timely medical help.

Structural factors related to the health services such as the long process of admission [11], [24], and challenges with referral systems resulted in frustration and fatigue in women when following their treatment [11]. In addition, medical error and the assurances which some women received from their physician involved patient delay in seeking medical help [11], [26], [29], [31].

Unpleasant previous experiences related to health service providers [11] and women’s trust in the knowledge and skills of the physicians, were identified as factors affecting women’s help seeking behavior [23], [24], [25]. The non-delaying women appeared to have more confidence in their GP to solve their problem [24]. Moreover, some women had concerns about issues like unnecessary presentation, wasting the time or bothering the physician [6], [23], [24], [26], which were barriers to receiving care. Women’s preferences to be visited with a female GP [25] and the shame and embarrassment of breast examination as a private organ were reported in some studies, especially in China [12] and older women in Taiwan [28].

### Personal-environmental factors

Personal-environmental factors such as women’s socioeconomic status and their cultural beliefs influenced individual health seeking behaviors. There was limited evidence about influence of sociodemographic variables such as age, marital status, and educational level on help seeking behaviors in the included studies. However, women who had lower socioeconomic status [11] or had recently immigrated [12] were more likely to delay the seeking of help. Indeed delayed presentation was apparent in older Chinese [12] and Taiwanese women [28].

However, the influence of women’s knowledge on help seeking behavior was obvious in the majority of the included studies. Lack of specific knowledge about breast cancer symptom as well as its risk factors and screening modalities was identified as an important influencing factor on delayed presentation in studies from different nations including Mexico [11], China [12], Malaysia [30], Ethiopia [29], Iran [31] and Taiwan [28].

General and specific beliefs about disease and breast cancer, such as a general belief like, “if your body has no pain, it means that there is no injury” in Mexican women [11] or “a wound which opens up heals by itself, little by little” in Iranian women [31] or “if a lump ain’t bothering you, you shouldn’t bother it. It will probably go away” in black women of eastern North Carolina [32], caused ignoring early or late signs of breast cancer.

There were also limited data to discuss the effects of optimism, curability, spirituality and fatalism on help seeking behaviors. However, optimism related to the nature of the symptom or the outcomes of breast cancer had different consequences. Optimistic beliefs related to the nature of symptoms directed the interpretation of symptoms towards less serious labeling, [30], while positive attitudes regarding the outcomes of cancer prompted help seeking [25].

It is noteworthy that women’s views about the curability of breast cancer varied among different societies. Fatalistic beliefs were not pervasive in Ireland [25], whereas conversely fatalistic views were common in Malaysian women [30] and black women of eastern North Carolina [32] It is notable that curability was related to prompt help seeking and a fatalistic view was linked to delayed help seeking [25], [30], [32]. There was a tendency to express a fatalistic view of cancer in relation to prior negative experiences in the family (25, 30] and cultural beliefs about cancer [32].

Spiritualty and its effect on help seeking behavior has been noted in some studies. African American women viewed “spiritual care as a form of self-care” and a “mission to get other women to seek treatment early” [27]. Spirituality also helped African American women to deal with stress and uncertainty following a primary diagnosis of breast cancer [27]. Conversely fatalistic view of cancer led to yielding to God as the only source of power to fight the cancer rather than acceptance of a biomedical treatment among some of the black women of eastern North Carolina [32]. Mahoney et al. (2011) also showed that although religious beliefs had a positive effect for some women in Ireland, one woman who relied on her faith had delayed seeking help [25].

## Discussion

The results of this study confirmed that meta-ethnography is a possible and useful method for synthesis of qualitative studies. More recent focus on help seeking behavior led to identification 12 papers related to the 2000–2013; in contrast only one paper related to the 1990–1999 years. The synthesis of 13 qualitative studies led to the identification of eight key concepts that influenced help seeking behavior of women with self-discovered breast cancer symptoms including symptom detection, initial symptom interpretation, symptom monitoring, social interaction, emotional reaction, priority of seeking medical help and emotional reactions, priority of seeking medical help, appraisal of health services and personal-environmental factors.

### Symptom detection

Symptom detection was the initial step in the help seeking process. The present study shows that in most cases, breast lumps were detected by chance or following occurrence of pain despite awareness of some women of BSE and performing it regularly. However, in a number of reviewed studies, there were some gaps in the reporting about how women detect symptoms. This finding is consistent with results from other studies in which women usually discover breast cancer symptoms outside of a structured breast self-exam while bathing or getting dressed [3]. Although there is no clear evidence to support the efficacy of BSE in decreasing breast cancer mortality [39], “breast awareness is still important” [40]. Research has shown that self-awareness is more effective than BSE [7], [39] and it appears that BSE training could provide an opportunity to increase women’s knowledge about breast cancer in developing countries [29], [41], [42].

### Initial symptom interpretation

This synthesis in the line of research on other types of cancer [43], [44] and diseases [45] propose symptom interpretation as the first and most important step in the help seeking process after symptom discovery. The present finding corroborates the ideas of Andersen who suggested that symptom appraisal constitutes 60%–80% of the total delay [46]. This study offered evidence that women initially tended to evaluate and attribute breast symptoms as a non-serious cause as seen in other types of cancer [43], [47]. In this regard, Scott et al. (2006) described that individuals who discovered potentially malignant oral symptoms, initially attributed their symptoms to less serious causes such as mouth ulcers and physical trauma [47]. Similarly, Ristvedt et al. (2005), showed that most patients with rectal cancer tended to initially attribute their symptoms to benign causes such as hemorrhoids [48]. Attributing symptoms to non-threatening causes contributed to delayed presentation. These results are consistent with multiple review articles conducted in common types of cancer including breast cancer [44], [49], [50].

Symptom interpretation is primarily based on elements such as comparing the nature of symptom with preexisting women’s knowledge and experiences regarding the breast disease symptoms. This finding has been similarly supported by research conducted on breast cancer with quantitative approach. These studies indicate that the nature of symptoms and patient knowledge can influence the timing of presentation; specifically, breast lumps are associated with fewer delays than symptoms that do not include lumps [8], [51], [52], [53]. Studies on common types of cancer also show that well-known warning signs of cancer such as lumps, pain, and bleeding are associated with fewer delays. Conversely, vague or atypical signs that could not be matched with patient knowledge and expectation about cancer may lead to a delay in seeking help [44], [49], [54].

The present study shows inconsistent results about the acknowledgment of breast lumps with or without pain as a well-known symptom of breast cancer. For some women, the presence of pain was reassuring and resulted in delayed presentation, whereas pain was the strongest trigger to promote action in the majority of included studies, particularly studies on Asian women.

Women’s perception of breast cancer risk also influenced symptom interpretation. The present study also showed that those women who considered themselves at low risk for breast cancer also attributed symptoms to less serious causes. Asian women were less likely than other women in this review to believe that they are at risk of breast cancer. A review study by Jones et al. (2014) demonstrated that African American women’s poor awareness about breast cancer, and their perception of being not at risk of breast cancer affected their sensitivity to breast cancer symptoms and subsequent health behavior [53]. Family history of breast cancer affected women’s perception of being at risk of breast cancer as it was seen in other studies [55], [56].

### Symptom monitoring

The initial interpretation of symptoms changed across the help seeking process, through monitoring of symptom progression and information seeking by social interactions. Scott et al. (2006) supported these findings through the description of symptom appraisal among patients with potentially malignant oral symptoms. They illustrated that initial interpretation of symptoms is revised over time, based on obtaining new information, persistence of symptoms, and symptom progression [47]. A qualitative synthesis in common type of cancer also showed that most patients monitored their symptom for worsening or developing additional symptoms to take action [44]. Indeed, some studies showed that some women who delayed presentation believed that their symptom would go away [57], [58], [59]. While some researchers suggested that this belief is indicative of denial [60], some challenged this view, and believed that women who initially dismiss the symptoms, not only deny but also actively monitor the symptom for worsening or developing additional symptoms [12], [23].

### Social interaction

Symptom disclosure was prevalent among women after discovering symptoms, and had a supportive role for timely help seeking. In this regard, studies with quantitative approaches showed that women who fail to disclose their symptoms with someone else are more likely to delay the seeking of help [8], [10], [59]. However, for some women, fear of bothering close relatives was the main reason for hiding the symptoms. A systematic review also showed that fear of stigma was the main reason for reluctance to disclose symptoms among black women in the USA and the UK [53].

Sanctioning of help seeking by significant others for example close relative or friends was important factor on early presentation. Review studies on common cancers have highlighted the role of significant others’ approval of help-seeking in prompt consultation [44], [49].

### Emotional reactions

Our finding was consistent with the prior studies [47], [60], [61], which showed that lack of worry about nature of the symptoms delayed seeking help. However, research does not suggest consistent evidence about effects of emotional reaction on help seeking behaviors and fear of cancer could contribute to both prompt [60] and delayed help seeking [51], [53], [58], [62].

In our review, negative emotional reactions such as denial, fear of cancer and the consequences of medical treatment denial were significant barriers to seeking help in countries with different cultural context including China [12], Iran [31], Taiwan [28], Malaysia [30], Ireland [25] as well as African American women, but fear of losing femininity was only reported in African American women [27]. A recent review (2014) reported concerns such as losing a partner and fear of treatment as important factors which prevent black women from seeking help [53]. This finding emphasizes the importance of sociocultural factors involved in delayed presentation of women with breast cancer symptoms.

### Priority of seeking medical help

Perceived severity of the symptoms could strongly encourage women to seek medical help, although some women did not prioritize their health over competing demands. Consistent with other studies [44], competing priorities were significant barriers to seeking help in different nationalities. However, caring behaviors for family members and others did not play a negative role in African American women. The latter finding was supported by a systematic review in black and African American women [53].

Moreover, some women who postponed seeking medical help had chosen alternative therapy as the first choice to deal with their problem. Taib et al. (2011) reported that some Malaysian women lacked confidence in conventional medicine and thus preferred alternative therapy [30]. Similarly, Lannin et al. (1998) reported that a large number of African American patients presenting with late stage breast cancer preferred to use herbal remedies as they were afraid of breast surgery and losing their partner’s support [63].

### Appraisal of health services

A number of included studies underscored the impacts of financial constraints and inaccessibility of healthcare on delayed help seeking. Consistent with other studies [53], [64], women’s trust in the knowledge and skills of the physicians were identified as factors affecting patients’ help seeking behavior. In our study, shame and embarrassment of breast examination were barriers to receiving care only in Chinese women and fear of wasting doctors’ time was only reported by non-Asian women. Similarly, according to a population-based survey among women from different ethnic groups in Eastern London, embarrassment and concerns about wasting the doctor’s time were barriers to seeking medical care by South Asian and white women, respectively [65].

Smith et al. (2005) and O’Mahony et al. (2009) have highlighted the role of patient’s gender as an important factor in help seeking behaviors [44], [66]. Smith et al. (2005) by synthesizing experiences of men and women for different types of cancer stated that “although men and women expressed embarrassment about consultations with doctors if symptoms affected a sexual area of the body, men especially feared weakness and loss of masculinity associated with seeking help” (44). In addition, O’Mahony et al. (2009) described that “help seeking is often viewed as non-masculine apart from situations in which men’s sexuality could be negatively affected by delayed treatment” (66).

### Personal-environmental factors

Our reviewed studies often did not provide adequate information about socioeconomic status and cultural factors that influence help seeking behaviors. Unger-Saldaña and Infante-Castañeda (2011) provided favorable description that “certain characteristics of the local health services, in combination with the patient’s contextual characteristics determine what kinds of service(s) are used” [11].

However, according a thematic review, research on the relationship between sociodemographic factors and patient delay has shown mixed results [67]. For example, Meechan et al. (2002) found no association between delay time and sociodemographic factors in breast cancer cases [68], in contrast, some other evidence consistent with our results shows that delayed help seeking was associated with older age [4], [69], ethnicity [52], [70], and lower socioeconomic status [52], [70].

The current study also showed that Asian women had limited knowledge and more fatalistic views about breast cancer. Public perception of the incurability of cancer was a reason for delay in Chinese, Malaysian, Iranian and Taiwanese women. Fatalism was reported as a major barrier to seeking medical help, especially in Asian and African women [63], [53], [71].

In this regard, Taib et al. (2011) discussed that because of a relatively low incidence of breast cancer and high incidence of patients with delayed presentation in Malaysia, women not only have low encounters with breast cancer patients but also encounter patients with advanced disease and poor outcome; thus, their experiences promote the perception of disease incurability [30]. Lannin et al. (2002) reviewed the reasons of racial differences in breast cancer mortality in African American women, and stated that socioeconomic factors only in combination with cultural beliefs can explain the “mortality gap” in African American women [70]. Optimism and religiosity had conflicting effects on help seeking behavior. However, our finding was not completely conclusive.

## Conclusion

This study revealed that women’s knowledge and perception of being at risk of breast cancer influenced their initial interpretation. In the best-case scenario, women attributed the symptoms to a serious cause which required to be confirmed. They discussed the problem with others and were encouraged to seek medical help. In the worst-case scenario, on the other hand, a painless lump was perceived as not important. Women considered themselves to be at lower risk of developing breast cancer and received reassuring comments (based on the “wait and see” approach) when they disclosed their symptoms to others. In addition, their social experiences and beliefs implied the stigma, incurability, and severe internalized fear of cancer. Competing priorities, financial constraints, the high cost of healthcare, previous negative experiences with healthcare, fear of cancer diagnosis, and embarrassment of physical exams reinforced their doubts and delayed their presentation. The latter scenario was more compatible with narratives of Asian women.

## Implication

The findings of this review has implications for effective intervention in order to shorten patient delay. Sociocultural and health care system characteristics dictate specific needs to develop interventions to reduce patient delay. Symptom interpretation is mainly influenced by women’s scientific knowledge about breast cancer. Therefore, public educational programs have to focus on increasing women’s awareness about all symptoms, risk factors, and cost-effective screening programs of breast cancer in a clear and understandable manner. Burgess et al. (2001) described that “educational messages would need to be designed with some care so as not to cause undue alarm among women and an overload of referrals and demands for consultations in both primary and secondary care” [23]. Our findings also emphasized the importance of social interaction in providing new information and sanctioning of help seeking. Hence, women should be encouraged to discuss their symptoms with other individuals. Indeed public education on how to advise others in these situations is also warranted. As fear and stigma of breast cancer and fatalistic view caused delayed help seeking, women need to be informed about early detection programs and their promising results.

Delivering telephone counseling by professionals as a convenient, inexpensive, fast and without face to face confrontations can be a useful tool to inform and encourage symptomatic women to seek help especially for those with fear of embarrassment of clinical breast examination or occupational commitments. Also improvement of referral systems and decreasing the cost of services can be useful strategies to shorten patient delay.

## Limitations

The results of the present research were influenced by the approaches, strengths, and weaknesses of the reviewed studies. These results suggested sufficient information about the cognitive component (i.e. the role of women’s knowledge about breast cancer) of help seeking behaviors.

The studies included in this synthesis were conducted in different countries. They did not usually provide a detailed description of underlying factors such as socioeconomic and cultural beliefs. In addition, there were inadequate data about the characteristics of the studied healthcare systems. Therefore, there were insufficient data to justify the considerable difference in the length of delay between various studies. However, constant comparison of the concepts enabled us to recognize the significant differences and similarities rooting from contextual factors. Nevertheless, qualitative studies are warranted to clarify how cultural beliefs affect health and medical help-seeking behaviors.
